# Helminths, Ticks, and Calf Mortality in Ethiopia: A Prospective Cohort Study

**DOI:** 10.1155/vmi/1246269

**Published:** 2026-05-25

**Authors:** Moti Wakgari Amenta

**Affiliations:** ^1^ Jimma University College of Agriculture and Veterinary Medicine, P. O. BOX. 307, Jimma, Oromia, Ethiopia; ^2^ Bedele Regional Veterinary Laboratory Center, P. O. BOX. 15, Bedele, Oromia, Ethiopia

**Keywords:** calves, Ethiopia, helminths, mortality, ticks

## Abstract

There is a significant demand for animal products in Ethiopia, driven by the country’s rapid urbanization and population growth. In contrast, calf death has been hindering cattle productivity. This study investigated helminth and tick species affecting calves and evaluated whether calf mortality exceeded accepted management thresholds. The purposes of this study were to accurately identify tick and helminth species present in collected samples, to quantify morbidity and mortality rates, and to determine the prevalence of helminth infection and tick infestation. From January to December 2024, a prospective cohort study using a cluster‐randomised sampling design was conducted on calves aged up to 1 year. The study population was clustered into semi‐intensive and smallholder dairy farms. More than half (57.6%, 95% CI: 0.5287–0.6230) of the fecal samples tested positive for helminth parasite eggs of various species. Different tick species were observed on 92% (95% CI: 0.8912–0.9432) of the calves examined. Mortality rate was 15.08% and significantly associated with locations and higher in Bedele (OR = 2.9, *p* = 0.029, 95% CI: 1.21–7.04), Didessa (OR = 2.6, *p* = 0.037, 95% CI: 1.14–5.98), semi‐intensive production system (OR = 3.97, *p* = 0.015, 95% CI: 1.31–11.72), rainy season (OR = 4.8, *p* = 0.008, 95% CI: 2.39–10.56), and greater herd numbers (OR = 5.62, *p* = 0.001, 95% CI: 2.72–12.25). The projected crude mortality rate was high, and awareness‐raising should be undertaken to reduce the social and economic impact on owners’ livelihoods. All stockholders should implement strategic deworming with broad‐spectrum anthelmintics to control helminth species and use effective acaricides to control tick infestations.

## 1. Introduction

Over the next 3 decades, Ethiopia’s population is expected to increase significantly, rising from approximately 132 million to more than 190 million. This fast demographic expansion is expected to be accompanied by accelerated urbanization, with both population growth and the rate of urban development reaching historically high levels during this period [1–3]. Also, the demand for livestock and livestock products in Africa between 2030 and 2050 will increase two‐ to eight‐fold [4]. The nation’s annual production of cattle and livestock products, such as milk, meat, and eggs, is inadequate to meet the demands of the growing human population both now and in the future. The current livestock production system needs to be changed to one focused on the commercial market to meet government goals of reducing poverty, guaranteeing food security, improving nutrition, generating foreign exchange, and boosting the national economy [5].

The main factors influencing livestock performance in Ethiopia are inadequate feed, common infections, low‐quality breeding stock, and weak regulations for credit, extension, marketing, and infrastructure [6]. Moreover, a high rate of calf illness and mortality results in substantial financial losses for dairy farmers, including lost output throughout the calf’s life, medical expenses, restricted dairy herd growth, and reduced genetic selection [7].

To have healthy calves, dairy calves must be raised with care in three areas: a clean, healthy calving pen, early intake of high‐quality and sufficient volume of colostrum, and good dam health. In the global cattle‐rearing industry, calf morbidity and mortality are issues [8], yet several factors may explain why tropical areas have higher rates of these conditions than temperate areas. Several infections that can harm calf and heifer performance through disease flourish in the tropical climate. The high humidity and temperatures in tropical climates facilitate the multiplication and spread of pathogens, which is a problem for the health of newborn calves [9].

In Ethiopia, the primary causes of death in dairy calves are diarrhea, pneumonia, and septicemia [10]. Globally, gastrointestinal parasites that can infect cattle include nematodes such as strongyle species (*Haemonchus*, *Ostertagia*, *Trichostrongylus*, and *Cooperia*), as well as trematodes of economic significance. Along with cestodes like *Moniezia* species (*Moniezia benideni* and *Moniezia expanza*), *Fasciola* species (*Fasciola hepatica* and *Fasciola gigantica*) and *Paramphistomum* species (*Paramphistomum cervi*) could also be significant barriers to animal production [11–16]. Particularly in young animals, the expected mortality rate is around 10%, and weight loss of 6–12 kg per animal per year is possible [17].

Around 47 tick species are present in Ethiopia, most of which are important as vectors and disease‐causing agents and have a damaging effect on skin and hide production [18, 19]. Ticks cause substantial losses in cattle production, in terms of diseases, reduced productivity and fertility, and often death, and are economically the most important ectoparasites of cattle [20]. They are major vectors of many animal diseases, posing emerging economic and health problems in tropical and subtropical countries worldwide [21].

The two major obstacles to increasing Ethiopia’s periurban and urban dairy output are crude calf illness and mortality. According to reports, yearly calf mortality in periurban and urban dairy production systems ranges from 15.3% to 25% [22]. In a similar vein, an extensive livestock production system in Southwestern Ethiopia reports 34.90% morbidity and 12.30% death [15]. Also, recently, other authors reported calf mortality rates of 12.85%, 12.97%, and 15.2% [10, 12, 23].

Gastrointestinal helminths and ticks are among the main constraints affecting cattle production in Ethiopia, mainly in young calves. Numerous studies have reported the prevalence of helminth parasites and tick infestations in cattle in different parts of the country. But most of these studies focused mainly on adult cattle or assessed either internal parasites or ectoparasites separately. Also, information on the prevalence of helminth parasites and tick species affecting calves under 1 year of age remains limited in many areas of Ethiopia. Therefore, generating baseline information on major helminth parasites and tick infestations in calves is crucial for designing appropriate prevention and control strategies. There has been limited information from prospective cohort studies following calves from birth to 1 year of age in Ethiopia [15, 16]. Even though many studies indicate that calf health problems remain a major constraint to dairy productivity, they mainly focus on the magnitude of mortality and morbidity and provide limited information on identifying helminth and tick species, particularly in calves up to 1 year of age and in specific local production systems. Therefore, the following goals were suggested for this study:

• To identify major helminth parasites and tick species.
• To quantify crude morbidity and mortality rates of calves.
• To determine the prevalence of helminth parasite infection and tick infestation.

## 2. Materials and Methods

### 2.1. Description of Study Area

**FIGURE 1:**
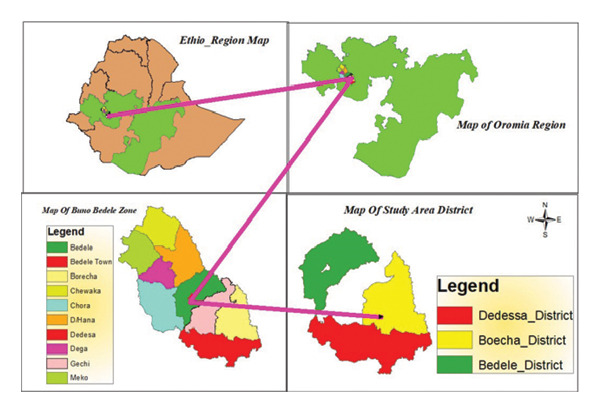
Map of study area.

The study was conducted in the Bedele, Borecha, and Didessa districts of the Buno Bedele zone, Ethiopia. Buno Bedele zone is located at 488 km from Addis Ababa in the southwest of Ethiopia (Figure 1). This zone has an area that covers 5964 Km^2^ (596,400 ha) and is located between 7° 58′ 35″N–903′ 7″ N latitude and 35° 51′2″ *E* − 36,051′32″ *E* longitude. It has four seasons: the summer season with heavy rainfall, the autumn season, the winter season, and the spring season, which has a light rainy season. It has an average annual rainfall of 1800–1900 mm and an altitude of 1310–2380 m; temperature varies from 11.3°C to 28.5°C [24].

### 2.2. Study Population

The study population comprised all local and crossbreed calves up to 12 months of age living in randomly chosen nine peasant associations and households of purposively selected districts. The calves were categorized by weaning age (less than 6 months and 6–12 months), sex (male and female), body condition (optimum and thin) [25], and breed (cross and local). Other risk factors include production system (semi‐intensive and smallholder), mixing different age groups (no and yes), cleaning activity (irregular and regular), and season (rainy and dry). Crossbred calves are those that have blood from both exotic (Jersey and Holstein) and local breeds (Zebu). Stillborn and congenitally malformed calves were excluded from follow‐up, and extra emergency visits were made as required for health issues. Only calves up to 1 year at the beginning of follow‐up were included in this research. The health problems observed during regular visits were assessed through clinical examination, whereas the farm attendants were asked to list and describe any health problems that emerged between visits. Sick calves within the age category were included as morbidity and mortality from the day of follow‐up onward.

### 2.3. Data Collection

To quantify calf morbidity and mortality, a semistructured questionnaire survey and different formats were used during follow‐up. A pilot test of the questionnaire was carried out with a small sample of participants not included in the final study, which helped assess the clarity, comprehension, and feasibility of the questions. Feedback from the pilot has led to further refinement of some points to increase clarity and reduce ambiguity.

### 2.4. Study Design

It was conducted from January to December 2024 as a longitudinal study using a design that involved eight owners of semi‐intensive dairy farms and 105 smallholder farmers, for a total of 113 owners, to quantify calf mortality. Calves from selected owners aged up to 1 year were followed monthly, and appropriate morbidity, mortality, and other data were collected. To identify helminth and tick species and determine their prevalence, fecal and tick samples were collected from February to April and submitted to the laboratory.

### 2.5. Sampling Technique and Sample Size Determination

Since there was no prior research in the study area, an expected animal‐level prevalence of 50% and a targeted absolute precision of 5% with 95% CI, along with cluster random sampling, were used [26]. First, districts were purposively selected, while Kebele or puissant associations were selected randomly. Due to the large and dispersed population of smallholder dairy farms, single‐stage cluster sampling was selected, which was mutually exclusive, collectively exhaustive and heterogeneous within clusters. The study population in the selected Kebeles was clustered into semi‐intensive and smallholder dairy farms. There were 1050 smallholders and 80 semi‐intensive dairy farms in selected Kebeles. This study included 105 smallholders and 8 semi‐intensive randomly clustered dairy farms. The average sampling unit per cluster from both farming systems was 3.7 (418/113 = 3.7).

(1) n=z2×p1−pDEFFd2,n=1.962∗0.51.0941810.5−∗=0.05.

DEFF = 1 + (cluster size − 1)∗ICC, ICC = intraclass correlation, and DEFF = design effect, where *n* is the sample size, *z* is the statistic that describes the level of 95% confidence interval, *p* is the expected prevalence, *q* is the expected nonprevalence (1 − *p*), *d* is the relative required precision, and DEFF is the design effect of 1.09. As a result, 418 calves of both local and cross breeds were sampled.

The average sampling unit per cluster from both farming systems was 3.7 (418/113 = 3.7).

(2) DEFF=110.0334+m−1∗ICC=+3.71−∗=12.70.0334+∗=1.09.

The intraclass correlation was 0.0334, which was calculated from the variance between clusters and within clusters. *m* = average number of sampling units.

(3) ICC=σ2 betweenσ2 between+σ2 within.

Between cluster variance (*σ* ^2^between) = 0.0334, within cluster variance (*σ* ^2^within) = 0.966, and *σ* ^2^ = variance.

### 2.6. Sample Collection and Transportation

#### 2.6.1. Sedimentation and Flotation Method

Using arm gloves, 418 fresh fecal samples were collected from the calves’ rectums. The fecal samples were transferred to the laboratory after being placed in universal screw‐capped bottles and preserved in 10% formalin. Standard sedimentation procedures were used to inspect fecal samples. The calf was considered positive if at least one parasite egg was detected in either sedimentation or flotation or both procedures.

#### 2.6.2. Tick Collection and Preservation

The chosen calves were first tightly held and observed for tick infestation. For identification purposes, ticks were manually removed using the hand from the udder, scrotum, ear, anus, vulva, perineum, dewlap, brisket, and belly and under the tail [27]. Calves were considered positive for the presence of one or more of the ticks found on calves. The collected ticks were submitted to the Bedelle Regional Veterinary Laboratory for taxonomic identification, where they were maintained in individual prefilled universal bottles containing 70% alcohol. The collected ticks were classified using a stereomicroscope into various genus and species levels [28].

### 2.7. Data Management and Analysis

Data gathered from the field were entered into a Microsoft Excel spreadsheet after being captured in record sheet format. Stata version 13 was used to perform a multivariable logistic regression analysis to examine the relationship between the outcome variable and the different explanatory variables, controlling for the possible effect of confounders. By dividing the total number of tested samples by the number of positive samples, and then multiplying the result by 100, the overall prevalence was determined. The odds ratio of 1 shows no association, while a value greater than 1 suggests a positive association between the exposure and the outcome. On the other hand, a value below 1 suggests a negative association. Using the Hosmer–Lemeshow test, a *p* value greater than 0.05 indicated a good fit, so the null hypothesis was accepted; with a *p* value less than 0.05, indicating a poor fit, the null hypothesis was rejected, showing a difference between the observed and predicted values [29]. An odds ratio (OR) > 1 indicates higher odds of the outcome (infestation, infection, or mortality) in the exposed group than in the reference group, while an OR < 1 indicates lower odds.

## 3. Result

### 3.1. Descriptive Epidemiology

#### 3.1.1. Prevalence and Major Identified Gastrointestinal Parasite Species

During the longitudinal study period, 418 calves participated in the research. About 241 (58%) of the fecal samples collected were positive for one or more helminth parasite eggs of various species (Table 1).

**TABLE 1:** Prevalence and major identified helminth parasite species.

Identified helminth parasite species	Number of calves infected by the parasite	Prevalence (%) 95% CI
*Paramphistomum spp*	53	22 (0.1722–0.2764)
*Fasciola spp*	45	18.7 (0.1426–0.2407)
*Ascaris spp*	32	13.3 (0.0956–0.1814)
*Oesophagostomum spp*	27	11.2 (0.0781–0.1581)
*Trichuris spp*	23	9.5 (0.0644–0.1391)
*Bunostomum spp*	18	7.5 (0.0478–0.1150)
*Strongyloides spp*	15	6.2 (0.0381–0.1001)
*Cooperia spp*	11	4.6 (0.0257–0.0799)
Mixed	17	7 (0.0445–0.1101)
	241	58 (0.528–0.623)

#### 3.1.2. Prevalence and Major Identified Ticks’ Genus and Species

From the collected ticks, three genera and three species were identified (Table 2).

**TABLE 2:** Prevalence of tick genus and species in the study areas.

Genus	Species of ticks	Prevalence (%)	95% CI
*Boophilus*	*B. decoloratus*	44 (272/618)	0.4015–0.4795
*Amblyomma*	*A. variegatum*	30 (185/618)	0.2646–0.3366
*Hyalomma*	*H. marginatum*	20 (124/618)	0.1710–0.2340

#### 3.1.3. Crude Morbidity and Mortality Rates

##### 3.1.3.1. Crude Morbidity Rate

Crude morbidity rate during the follow‐up period was 42.80% (179/418). Table 3 shows a slight variation in morbidity rates across locations.

**TABLE 3:** Crude morbidity rate.

Locations	No. of calves		No. of diseased calves				
			Age		Sex		Morbidity rate (95% CI)
	Total sick		< 6 months	6–12 months	M	F	
Borecha	129	45	27	18	19	26	34.88 (0.2720–0.4344)
Bedele	146	65	50	15	29	36	44.52 (0.3670–0.5262)
Didessa	143	69	41	28	31	38	48.25 (0.4022–0.5638)
Total	418	179	118	61	79	100	42.80 (0.3817–0.4761)

##### 3.1.3.2. Crude Mortality Rate

The crude mortality rate was 15.07%. There were a few differences in death rates among the locations, as shown in Table 4.

**TABLE 4:** Crude mortality rate.

Locations	No. of calves	Total death	No. of died calves				Mortality rate (95% CI)
			Age		Sex		
			< 6 months	6–12 months	M	F	
Borecha	129	12	9	3	4	8	9.3 (0.0540–0.1556)
Bedele	146	23	16	7	9	14	15.75 (0.1073–0.2253)
Didessa	143	28	20	8	10	18	19.58 (0.1391–0.2684)
Total	418	63	45	18	23	40	15.07 (0.1196–0.1882)

### 3.2. Prevalence and Associated Risk Factors of Helminth Parasites

The Bedele and Didessa locations were 1.4 and 1.2 times more likely to have the infection, respectively, than the Borecha location, and there was a significant association between helminth infection across districts (*p* = 0.005,  0.017). Thin body condition was 1.85 times more likely to have the infection than optimum body condition, which was statistically significant (*p* = 0.004) (Table 5).

**TABLE 5:** Risk factors associated with helminth parasites.

Variables	No. of calves examined	No. infected	Prev (%)	OR (95% CI)	Chi‐square value	DF	*p* value
*Locations*							
Bedele	146	92	63	1.4 (1.12–1.85)	11.24	2	0.005
Didessa	143	81	56.7	1.2 (1.05–1.53)			0.017
Borecha	129	68	52.7	RF			RF
Total	418	241	57.6	(0.5287–0.6230)			

*Body condition*							
Thin	213	131	61.5	**1.85** (1.21–2.83)	13.56	1	0.004
Optimum	205	110	53.7	RF			RF

*Age*							
6–12 months	243	140	57.6	1.02 (0.67–1.55)	1.45	1	0.919
< 6 months	175	101	57.7	RF			RF

*Sex*							
Female	236	138	58.5	0.76 (0.50–1.16)	2.73	1	0.207
Male	182	103	56.6	RF			RF

*Breed*							
Cross	75	43	57.3	0.94 (0.53–1.65)	2.14	1	0.828
Local	343	198	57.7	RF			RF

Abbreviations: CI, confidence interval; DF, degree of freedom; RF, reference factor.

### 3.3. Prevalence and Associated Risk Factors of Ticks

In the study districts of the Buno Bedele zone, 418 calves observed were positive for 1 or more tick species (92.1%). In Didessa, Bedele, and Borecha, the tick prevalence was 92.3%, 91.8%, and 92.2%, respectively. Thin body condition was 4.07 times more likely to have infestation than optimal body condition, which was statistically significant (*p* = 0.035). Local calf breeds were 2.7 times more likely to have an infestation than cross breeds, which was statistically significant (*p* = 0.001) (Table 6).

**TABLE 6:** Risk factors related to tick prevalence.

Variables	No. of calves examined	No. calves infested	Prevalence	OR (95% CI)	Chi‐square value	DF	*p* value
*Locations*							
Didessa	143	132	92.3	1.03 (0.50–2.12)	1.76	2	0.927
Bedele	146	134	91.8	0.83 (0.41–1.73)			0.964
Borecha	129	119	92.2	RF			RF
Total	418	385	92.1	(0.8912–0.9432)			

*Body condition*							
Thin	205	198	96.60	**2.7** (1.35–5.46)	20.58	1	0.035
Optimum	213	187	87.80	RF			RF

*Age*							
6–12 months	243	225	92.30	1.71 (0.67–4.32)	3.24	1	0.257
< 6 months	175	160	92.59	RF			RF

*Sex*							
Female	236	220	93.22	1.78 (0.72–4.37)	2.93	1	0.205
Male	182	165	90.7	RF			RF

*Breed*							
Local	343	333	97.10	4.07 (2.14–9.20)	37.29	1	0.001
Cross	75	52	69.30	RF			RF

Abbreviations: CI, confidence interval; DF, degree of freedom; RF, reference factor.

### 3.4. Risk Factors Associated With Calves’ Mortality

During the follow‐up, 21.24% (24/113) of calf owners experienced one or more calf deaths, with the highest rate (25%) in Didessa, followed by Bedele (19.44%) and Borecha (14.3%). Didessa and Bedele locations were 2.6 and 2.9 times more likely to have calf mortality than the Borecha location, respectively, which was statistically significant (*p* = 0.037, *p* = 0.029). Semi‐intensive production systems were 3.97 times more likely to experience calf mortality than smallholder production systems, a difference that was statistically significant (*p* = 0.015). Herds with more than three calves were 5.62 times more likely to have calf mortality than herds with one to three calves, which was statistically significant (*p* = 0.001) (Table 7).

**TABLE 7:** Risk factors associated with calves’ mortality.

Variables	No. of the owners	Owners who encountered calf deaths	Prevalence (%)	OR (95% CI)	Chi‐square value	DF	*p* value
*Location*							
Didessa	44	11	25	2.6 (1.14–5.98)	17.33	2	0.037
Bedelle	36	7	19.44	2.9 (1.21–7.04)			0.029
Borecha	42	6	14.3	RF			RF
Total	113	24	21.24				

*Production*							
Semi‐intensive	8	4	50	3.97 (1.31–11.72)	32.51	1	0.015
Smallholder	105	50	47.62	RF			RF

*Mixing different ages*							
No	63	31	49.20	0.68 (0.27–1.75)	2.63	1	0.434
Yes	50	23	46	RF			RF

*Cleaning activities*							
Irregular	60	29	48.30	1.16 (0.44–3.08)	1.65	1	0.759
Regular	53	25	47.20	RF			RF

*Weaning age*							
> 6 months	67	32	47.76	0.8 (0.34–2.32)	1.54	1	0.88
≤ 6 months	46	22	47.82	RF			RF

*Seasonal mortality*							
Rainy season	85	41	48.23	4.8 (2.39–10.56)	37.79	1	0.008
Dry season	28	13	46.43	RF			RF

*No. of calves at herd level*							
> 3	52	30	57.80	5.62 (2.72–12.25)	42.81	1	0.001
1–3	61	24	39.34	RF			RF

Abbreviations: CI, confidence interval; DF, degree of freedom; RF, reference factor.

## 4. Discussion

The present study’s overall helminth and tick prevalences were remarkably high, indicating that calves in the study area had significant levels of helminths and ticks. That might be the result of poor husbandry practices and a lack of social understanding concerning the effects of ticks and helminth parasites. The crude mortality rate was high, and this was evaluated to determine whether calf mortality exceeded accepted management thresholds [30]. The whole helminth prevalence in this study was 57.60%, which was consistent with findings of Dosa et al. [16], who showed a prevalence of 56.23%; however, this was higher than the findings of Amenta [15] and Desie et al. [14], who determined prevalences of 43.9% and 38%, respectively, and lower than the 79.1% reported by Tegegn et al. [31]. In the present findings, the helminth parasite species were *Paramphistomum spp*., *Fasciola spp*., *Ascaris spp*., *Oesophagostomum spp*., *Trichuris spp*., *Bunostomum spp*., *Strongyloides spp*., and *Cooperia spp*. which were consistent with the findings of Dosa et al. [16]. Few differences in prevalence and species may be due to management and husbandry practices, climate, accessibility to veterinary clinic services, owners’ educational level, study type and duration, and other factors.

The helminth parasite showed significant differences among locations, consistent with the findings of Desie et al. [14], who reported significant differences in helminth infection across different study areas. The difference may result from different husbandry practices, herd sizes, or levels of deworming expertise and awareness. According to the current study, the prevalence of helminth infection indicates that decreased body condition in calves was associated with helminth infection. This result was consistent with research conducted in Ethiopia [14, 32].

The total tick prevalence in the current study was extremely high and consistent with the findings of Nigatu and Teshome [33], who reported a total prevalence of 89.4%, but disagreed with the finding of Wegi [34], who reported an overall prevalence of 41.8%. Among the ixodid ticks collected, *Boophilus* (*B. decoloratus*) was the most abundant, followed by *Amblyomma* (*A. variegatum*) and *Hyalomma* (*H. marginatum*), which disagrees with Wegi [34] on species abundance, who reported *Amblyomma spp* was the most abundant, followed by *Boophilus spp* and *Hyalomma spp*. However, both studies agree on the types of species found. Numerous reasons, such as the lack of improved husbandry techniques, the inadequate use of veterinary services, climate, and study methodology, and the farmers’ lack of knowledge about the impact and management of ticks on calves, play a significant role in the widespread infestation and prevalence difference of ticks in calves [15, 35].

The existing investigation suggests that infestation is associated with calves’ body condition. This result is consistent with research showing that calves with thin body condition had higher infestation rates [15, 36]. Calves with thin body condition often have lower immunity, making them less able to resist parasite attachment, and weaker calves are less able to groom. Also, thinner calves have more delicate skin, which facilitates the penetration of tick mouthparts during feeding [15, 37].

The frequency of tick infestation varied significantly between breeds, which agrees with some researchers who found that tick infestation was more prevalent in local than crossbreed calves [15, 38]. Since most crossbreed animals are raised in urban rather than rural settings, there may be differences in owners’ understanding of the impact of ticks on their calves. Additionally, the proximity of veterinary clinic services and husbandry activities varies between towns and rural areas [15, 38].

The assessed crude morbidity and mortality rates in the current investigation were 42.79% and 15.08%, respectively. This is consistent with the morbidity rate and crude mortality rate of 40.29% and 12.85%, respectively, reported in Ethiopia by Rahmeto et al. [12], crude mortality of 16% reported in Kenya by Gurmu et al. [39], and crude morbidity and mortality rates of 49.5% and 15.2%, respectively, reported in Ethiopia by Abebe et al. [23]. Additionally, the coincidence with calf mortality ranged from 9.4% to 14% in mixed crop‐livestock production and from 15% to 25% in dairy production in urban and periurban areas in Ethiopia, according to Gebreyohanes et al. [5], and from 12.97% reported in Ethiopia by Ahmedin and Assen [10]. On the other hand, these figures are greater than the 10% and 6.49% yearly calf morbidity and mortality rates reported by Fentie et al. [22] in Ethiopia and 8.5% mortality rate reported in Botswana by Mosalagae et al. [40]. A significant mortality difference was observed among study areas or locations, consistent with the authors’ findings from Botswana by Mosalagae et al. [40]. The difference may arise from variations in husbandry practices, societal knowledge and awareness, accessibility to veterinary clinic services, study designs, production systems, and weather.

In this research, there was a significant difference in mortality between production systems, consistent with the findings of Yitagesu et al. [8, 15, 41]. The difference may arise from variations in husbandry practices, stocking density, increased disease transmission in higher population density, and environmental stress.

In this study, season had an impact on calf mortality, with the rainy season showing much higher mortality rates than the dry season, consistent with findings of other authors [40, 42, 43]. This may be explained by numerous interrelated factors. Rainy seasons are frequently associated with increased microbial load in the environment, a higher prevalence of infectious diseases, and reduced hygiene due to wet, muddy conditions. On the other hand, calf morbidity and mortality did not significantly change between the rainy and dry seasons [41].

Furthermore, greater herd size was associated with a higher calf mortality rate, consistent with the findings of Mellado et al. [44]. This study’s substantial association between calf mortality and herd size may be due to increased competition for feed and water in larger herds, as well as a higher incidence of disease and greater disease transmission in overcrowded herds.

The findings of this study provide information for veterinary extension and parasite control policies that emphasize community‐based, integrated management of helminths and ticks through routine deworming and acaricide administration [45, 46].

### 4.1. Research Limitations

Laboratory investigation of other microorganisms like bacteria, viruses, protozoans, and fungi that may play a significant role in the morbidity and mortality of calves was not being considered in this study. The load of helminth and tick parasites was not considered in this research. The morbidity and mortality caused by helminth parasites and ticks in calves were unknown in this study.

## 5. Conclusion

The combined high prevalence of helminth infections and tick infestations, along with the most identified helminth species such as *Paramphistomum*, *Fasciola*, and *Ascaris*, as well as ixodid ticks such as *B. decoloratus*, *A. variegatum*, and *H. marginatum*, cause direct and indirect production losses. These issues lead to reduced weight gain, growth retardation, skin damage, immunosuppression, and annoyance, while also serving as biological vectors for various bacteria, viruses, and protozoa, making them economically important. The estimated crude morbidity and mortality rates were high, and awareness should be raised to mitigate the social and economic impact on the livelihoods of owners. Consequently, all stakeholders should focus on strategic deworming with broad‐spectrum anthelmintics to control helminth species and use effective acaricides to manage tick infestations. The findings of this manuscript highlight the importance of helminth and tick species control to sustain calves’ health in addition to the quantified magnitude of calf mortality. This helps policymakers prioritize parasite control strategies, promote community awareness, and ensure the affordability of broad‐spectrum anthelmintics and acaricides to reduce economic losses and improve the livelihoods of livestock‐reliant communities.

## Author Contributions

Moti Wakgari Amenta: conceptualization; investigation; methodology; resources; project administration; supervision; formal analysis; writing–review and editing; software; and writing–original draft.

## Funding

Funding was not available for this project.

## Disclosure

The manuscript was previously posted as a preprint titled “Prospective Longitudinal Study of Calf Mortality: Prevalence and Identification of Helminths and Tick Species in Buno Bedele Zone Southwest Ethiopia” (DOI: 10.21203/rs.3.rs‐6463108/v1). I warrant that this research article has not been published and is not under consideration by another publisher.

## Ethics Statement

A formal letter (Ref. No. BRVL/S/38/17 on 05/01/2024) for this research was granted from Bedele Regional Veterinary Laboratory Centre, Department of Surveillance.

## Consent

The owners of the calves in this study provided informed consent prior to the study. Before commencing this research, I discussed with calf owners, local veterinary professionals, and government officials on types of samples, data, animal welfare, and the importance of this research.

## Conflicts of Interest

The author declares no conflicts of interest.

## Data Availability

Data supporting this study are available from the corresponding author upon reasonable request.
